# Holmium Complex with Phospholipids as ^1^H NMR Relaxational Sensor of Temperature and Viscosity

**DOI:** 10.3390/molecules27196691

**Published:** 2022-10-08

**Authors:** Olga Yu. Selyutina, Sergei P. Babailov

**Affiliations:** 1 Institute of Chemical Kinetics and Combustion, Institutskaya St. 3, 630090 Novosibirsk, Russia; 2 Institute of Solid State Chemistry and Mechanochemistry, Kutateladze St. 18, 630128 Novosibirsk, Russia; 3 A. V. Nikolaev Institute of Inorganic Chemistry, Siberian Branch of the Russian Academy of Sciences, Av. Lavrentyev 3, 630090 Novosibirsk, Russia

**Keywords:** lanthanides, lipid membrane, NMR, membrane viscosity sensor, paramagnetic shift

## Abstract

The sensitivity of Ho–phospholipid complexes to changes in the membrane viscosity of liposomes was checked. An increase in viscosity was observed for DPPC and DMPC near the phase-transition temperature. Ho–phospholipid complexes could be used as sensors of local membrane viscosity in NMR and MRI technologies.

## 1. Introduction

The paramagnetic properties of lanthanides are widely used in NMR, including biomolecular NMR and MRI. In biomolecular NMR, lanthanide ions provide an opportunity for determination of structures of protein–ligand complexes [1]. The existence of a hyperfine interaction between the unpaired electrons of a paramagnetic compound and the observed nucleus induces a paramagnetic shift—a paramagnetic relaxation enhancement—and it is an additional source of substantial broadening due to effects in bulk magnetic susceptibility [2,3,4,5]. Among other things, recent studies have demonstrated that coordination complexes of lanthanides could be sensitive to the viscosity of the media [6]. Studying paramagnetic-chemical lanthanide-induced shifts (LISs) and the bandshape of NMR signals for Ln complexes in solutions—as a function of the temperature—make it possible to obtain valuable information on their molecular dynamic and paramagnetic properties [7,8]. Lanthanide complexes with organic compounds could be used as NMR and MRI sensors for both temperature and pH [9,10,11]. In addition, it has been shown that complexes of lanthanides with phospholipids could be used as NMR temperature sensors [12,13]. It has been shown that complexes of thulium and praseodymium with phospholipids induce paramagnetic shifts, which have linear dependence on temperature and have features near the main phase-transition temperature of phospholipids [12,13,14].

Despite these features—which make lanthanide complexes promising as a temperature sensors for MRI applications—in biological systems, most of the data on the structure and dynamics of lanthanide complexes were obtained in homogeneous solutions [8,15,16,17,18]. There are significantly fewer studies of lanthanide complexes in heterogeneous systems, in particular those in lipid membranes. Thus, lanthanide complexes with phospholipids are very attractive from the point of view of their potential applications in medicine and biology.

Biomembranes form the boundary between the intra- and extracellular environment. The major component of biomembranes is the lipid bilayer. It is formed in water from phospholipids. The contents of the membrane influence the membrane’s properties, such as elasticity and viscosity which, in turn, influence the activity of membrane-bound proteins. Lipid bilayers exist in a fluid phase under physiological conditions, but can undergo phase transitions. Lipids can exist in different phases, including a liquid–crystal phase (L_α_), a gel phase (L_β_), and a ripple phase (P_β_). The main phase transition (*T_m_*) between L_α_ and L_β_ in a lipid bilayer changes the bilayer membrane structurally, from a less ordered state to a more ordered state, with changes in membrane fluidity. *T_m_* is affected by lipid headgroups structure and interactions as well as the number and position of unsaturated bonds in the fatty hydrocarbon chains, their length, and attachment to the headgroups. Phase transitions and lipid dynamics are intensively investigated in [19,20,21,22,23,24,25].

Saturated lipids and cholesterol could form so-called lipid rafts—with more rigid structures than the remaining lipid media—in membranes [26,27,28]. Therefore, determining the local viscosity in a cell membrane could be useful for studying the functioning of lipid rafts. Lipid rafts play important roles in many biological processes. Some membrane proteins are contained in lipid rafts, and these rafts can be a channel for the penetration of—for example—virus particles into a cell [29,30]. Additionally, intracellular viscosity abnormalities can lead to diabetes, neurodegenerative diseases, and cancer [31]. The use of lanthanide complexes could serve as a good method for determining tissue viscosity in MRI.

Liposomes, both multi- and unilamellar, are widely used as models of cell membranes. The simplicity of the model allows an understanding of specific interactions which are difficult to decouple in a cellular environment. Lanthanide ions have applications in clinical research including the treatment of burns, anticoagulants, antitumor therapy and imaging studies as well as acting as a tool for fundamental studies on model membrane systems [32]. For example, lanthanide-based shift-reagents are used to study the effects caused by lipid asymmetry in membranes [33,34].

The purpose of this study was to use examples of saturated and unsaturated phospholipids (DPPC, DMPC, and POPC) under different phase-transition temperatures to study the temperature dependencies of observed transverse relaxation times. According to the literature, a lanthanide-induced contribution to the relaxation rate enhancement is the sum of dipole–dipole, Fermi–contact, and Curie–spin components [17,35]. Additionally, the contribution of the Curie–spin component depends on the viscosity of the media and the temperature: $C\frac{\eta \left(T\right)}{T^{3}}$, where *C* is the constant, *T* is the temperature, and $\eta \left(T\right)$ is the dependence of the viscosity on the temperature [6]. According to analyses of the relaxation rates in some Ho complexes, the main contributions to the paramagnetic increase in the relaxation rates of the protons of ligands with characteristic distances from the paramagnetic center (4–6 A) are the Curie–spin and dipole–dipole contributions [36,37,38]. Moreover, the Curie–spin contribution at a magnetic field strength of 11.7 Tesla can reach more than 50% (in a temperature range of 273–330 K) in aqueous solutions. In this case, the dipole contribution to the paramagnetic increase in the relaxation rates is practically independent of temperature, while the Curie–spin contribution has a significant dependence on temperature.

**Figure 1:**
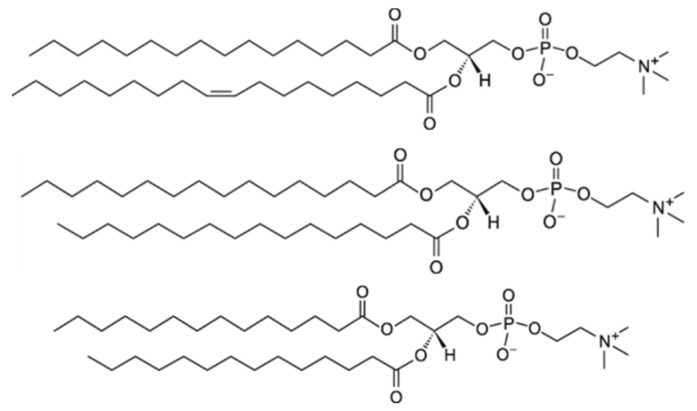
Structures of phospholipids: POPC (**top**), DPPC (**center**), and DMPC (**bottom**).

This work aims to show the fundamental possibility of using paramagnetic complexes of holmium and phospholipids as probes for temperature and viscosity in NMR, based on the model of POPC, DPPC, and DMPC liposomes (Figure 1).

## 2. Results

According to the literature, metal ions can effectively bind to phospholipids, forming a complex with 1–10 phospholipid molecules, depending on the valence and coordination number of the metal, as well as on the experimental conditions [39,40]. A carbonyl or phosphate group can act as a binding site in a phospholipid molecule [39,41]. In previous work, it was found that Ho–phospholipid complexes have stoichiometry of 1:3 in methanol solutions and complicated stoichiometry in water [14].

**Figure 2:**
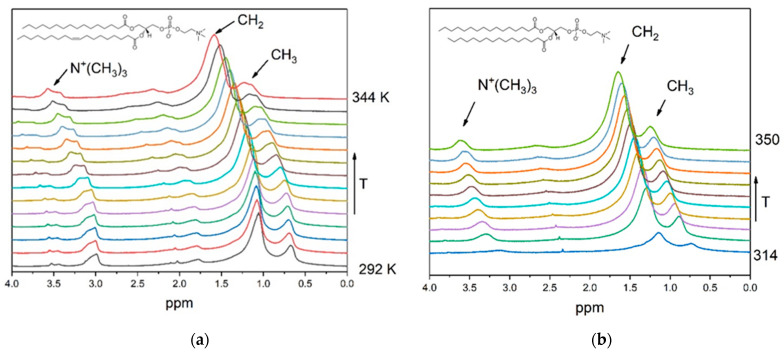
Fragments of ^1^H NMR spectra of (**a**) POPC and (**b**) DPPC liposomes at different temperatures.

Experiments were carried out for phospholipids with different phase-transition temperatures to observe differences in the responses of the Ho–phospholipid complexes in the systems with the different temperature dependences of the viscosity. Figure 2 shows fragments of ^1^H NMR spectra of POPC and DPPC liposomes at different temperatures. According to studies of bicelles, lanthanide ions bind with lipid phosphate groups [42]. Separation in the signal of the N^+^(CH_3_)_3_ groups is observed.

**Figure 3:**
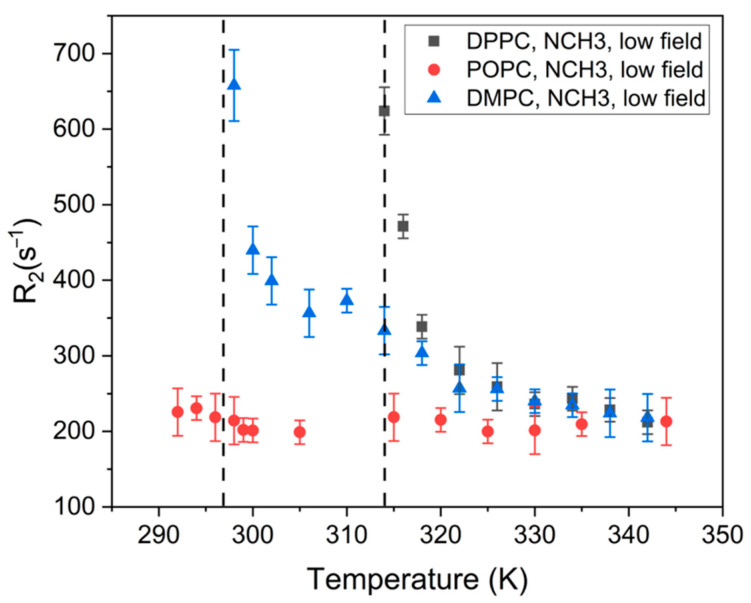
The dependence of the relaxation rate (*R*_2_) of the low-field components of the signal of N^+^(CH_3_)_3_ groups on the temperature.

**Figure 4:**
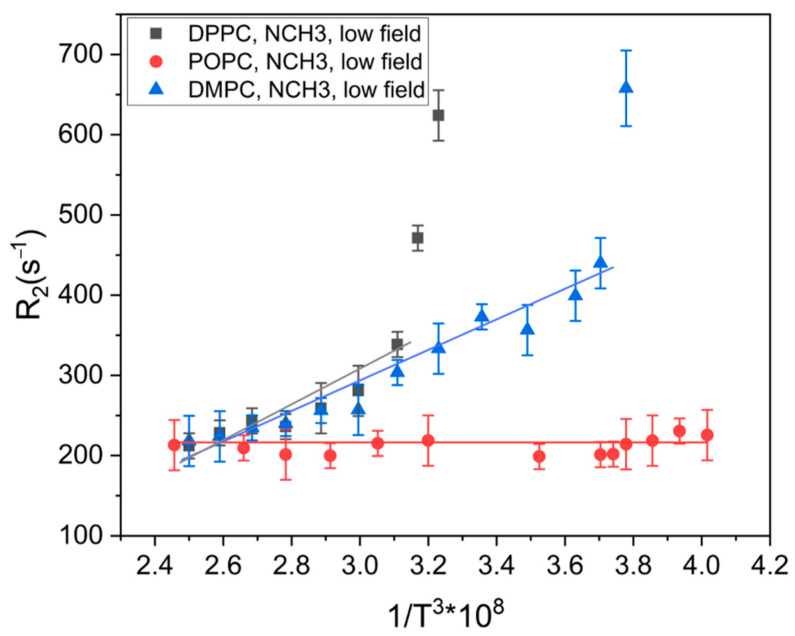
The dependence of the relaxation rate (*R*_2_) of the low-field components of the signal of N^+^(CH_3_)_3_ groups on the third power of the inverse temperature (1/*T*^3^).

Figure 3 shows the dependence of the low-field components’ relaxation rate (*R*_2_) in the signal of the POPC, DMPC and DPPC N^+^(CH_3_)_3_ groups on the temperature. According to the literature, a lanthanide-induced contribution to the relaxation rate enhancement is the sum of dipole–dipole, Fermi–contact, and Curie–spin components [17,35]. Additionally, the Curie–spin contribution depends on the viscosity of the media and the temperature: $C\frac{\eta \left(T\right)}{T^{3}}$, where *C* is the constant, *T* is the temperature, and $\eta \left(T\right)$ is the dependence of the viscosity on the temperature [6]. To estimate the dependence of the viscosity on the temperature for different systems, we plotted the dependence of the low-field components’ relaxation rate (*R*_2_) in the signal of the POPC, DMPC, and DPPC N^+^(CH_3_)_3_ groups on 1/*T*^3^ (Figure 4). It can be seen from Figure 3 that POPC does not have the temperature dependence of *R*_2_ or—correspondingly—the viscosity. A linear dependence of *R*_2_ on 1/*T*^3^ means that $\eta \left(T\right)$ is constant. The main phase-transition temperature of POPC is 270 K, and an observed effect could be caused by small changes in viscosity, which are beyond the sensitivity of the holmium complex in a temperature range of 292–344 K.

It should be noticed that significant difference is observed in the values of *R*_2_ of unsaturated POPC and saturated DPPC and DMPC while the values for DPPC and DMPC are close. The temperature dependencies of *T*_2_^*^ for DPPC and DMPC demonstrate similar behaviors in the temperature range 320–345 K; however, near the main phase-transition temperature (297 K for DMPC and 314 K for DPPC), the dependencies change dramatically. 

Linear dependencies of *R*_2_ on 1/*T*^3^ below the phase-transition temperature means that $\eta \left(T\right)$ is constant. It could be caused by small changes in viscosity, which are beyond the sensitivity of the holmium complex, same as in the case of POPC. However, near the phase-transition temperature, dramatic changes in transverse relaxation rate occur. It could be connected, with dramatic changes in the bilayer viscosity due to phase transition.

## 3. Materials and Methods

### 3.1. ^1^H-NMR

^1^H NMR spectra were recorded on a Bruker AVHD-500 (500 MHz) NMR spectrometer (Billerica, MA, USA) with temperature control. Deuterated solvent D_2_O (99.9% D, Aldrich, St. Louis, MO, USA) was used as-received. Relaxation rate (R_2_) was estimated using the signal line with in 1D NMR experiments. Analysis of the line shapes was carried out using Fityk software [43]. Due to the fast exchange between bound and free lipids, the half-width of the signal was associated with the spin–spin relaxation time, T_2_, through the following ratio:

R_2_ = 1/T_2_ = πW

Temperature control was achieved using a Bruker variable-temperature unit. All experiments were repeated three times.

### 3.2. Preparation of Liposome Samples

Liposomes were formed from 1-palmitoyl-2-oleoyl-glycero-3-phosphocholine (POPC, Avanti Polar Lipids (Alabaster, AL, USA), purity > 99%), 1,2-dipalmitoyl-sn-glycero-3-phosphocholine (DPPC, Avanti Polar Lipids, purity > 99%), and 1,2-dimyristoyl-sn-glycero-3-phosphocholine (DMPC, Avanti Polar Lipids, purity > 99%) (Figure 1).

Powder components were pre-dissolved in chloroform. After solvent evaporation, the dry lipid film was hydrated with D_2_O. The final concentration of lipid was 13 mM. The suspension was then sonicated (about 37 kHz, 1 h) to obtain unilamellar liposomes. NMR spectra were recorded for samples of 0.5 mL of vesicle suspension supplemented with 6 mM HoCl_3_ in 5 mm NMR tubes. Solvent peak was used as a reference.

## 4. Conclusions

Thus, in the present study, we studied the temperature dependencies of the effective transverse relaxation rate enhanced by lanthanide holmium in liposomes constituted from DMPC, DPPC, and POPC. The goal of the research was to show the limits within which holmium can be used as a probe. In the long term, nonlinear changes in the relaxation rates within the inflection region can be useful for practical use to determine the viscosity of infected tissues and to localize the focus of inflammation. The dependence of the relaxation rate on temperature opens up prospects for its use in measuring and plotting temperature in zones of inflammation, tumors, etc. For the correct use of lanthanides as temperature sensors, it is necessary to understand the nature of lanthanide-induced relaxation rate enhancement, and the features and limits of its applicability; this work provides answers to some of these questions.

The obtained data indicate that, in the POPC liposomes, with the main phase-transition temperature of 270 K, the viscosity of the bilayer does not depend on temperatures in the range of 292–344 K. For DPPC and DMPC, the liposomes bilayer viscosity does not depend on temperatures below the main phase-transition temperature (314 and 297 K, accordingly). It is likely that changes in the viscosity below the main phase-transition temperature of phospholipids are beyond the sensitivity of the holmium complex. However, near the main phase-transition temperature, there were dramatic changes in the viscosity of the bilayer for both DPPC and DMPC. In addition, significant differences were observed in the *R*_2_ values of unsaturated POPC and saturated DPPC and DMPC, while the values for DPPC and DMPC were close. This could mean that bilayer viscosity for saturated and unsaturated lipids is different.

Cell membrane viscosity plays an important role in many biophysical and biochemical processes, such as the diffusion of small biological molecules and membrane proteins, controlling the rate of chemical reactions, etc. Additionally, changes in the viscoelastic properties of cells could be the a marker of disease; for example, there are differences between the viscosities of tumor cells and normal cells [44]. Additionally, studying the local lipid bilayer viscosity is an important task for understanding cellular signaling pathways involving lipid rafts in particular [45]. Holmium–phospholipid complexes are sensitive to temperature due to the contribution of the Curie–spin component to the *T*_2_ relaxation rates. These complexes can be considered as relatively high sensitivity viscosity probes for in situ monitoring in phospholipid structures, such as liposomes or cell membranes.

## Data Availability

Not applicable.
